# Working conditions in a mental health institution: An exploratory study of professional nurses in Limpopo province, South Africa

**DOI:** 10.4102/curationis.v43i1.2081

**Published:** 2020-08-05

**Authors:** Ndivhuwo P. Mulaudzi, Ntsieni S. Mashau, Henry A. Akinsola, Tinotenda S. Murwira

**Affiliations:** 1 Department of Public Health, School of Health Sciences, University of Venda, Thohoyandou, South Africa

**Keywords:** high workload, mental health institution, mental healthcare user, inadequate resources, burnout, ineffective safety measures and professional nurses

## Abstract

**Background:**

Professional nurses are responsible for the provision of care, treatment and rehabilitation of all mental healthcare users (MHCUs) in the institutions for mental healthcare. However, professional nurses find themselves in difficult circumstances under which they must provide quality healthcare services to MHCUs.

**Objectives:**

The study explored and described the challenges experienced by the professional nurses working in a mental healthcare institution in Limpopo province of South Africa.

**Method:**

A qualitative approach was used to explore and describe the challenges faced by professional nurses working in a mental healthcare institution. The study was conducted from July 2016 to December 2016. Purposive sampling was used to select participants. Data were obtained through individual in-depth interviews with professional nurses between the ages of 26 and 50 years. Data collection continued until data saturation, which occurred after interviewing 18 participants. Tech’s open coding method was used to analyse data in this study.

**Results:**

Four themes emerged from data analysis, namely: inadequate safety measures, inadequate resources, impact of high workload and shortage of staff. The themes were further sub-divided into sub-themes.

**Conclusion:**

The study revealed several challenges that professional nurses face in mental healthcare institutions which might be a barrier to the provision of quality healthcare. Conducive working environments should be established to enable professional health nurses to provide quality nursing care, thereby promoting the health of MHCUs.

## Introduction and background

Nurses are at the forefront of admitting, treating and managing mental healthcare users (MHCUs) with different mental disorders in mental health institutions and are therefore exposed to different challenges in their work environments (Sobekwa & Arunachallam 2015:4). These nurses are confronted by a myriad of difficulties (Joubert & Bhagwan 2018:54) and significant challenges worldwide (Sobekwa & Arunachallam 2015:1). A study conducted in Palestine by Marie, Hannigan and Jones (2017:6) revealed that healthcare professionals were faced with a shortage of staff, which resulted in overworked nurses. A study conducted in Nigeria revealed that shortage of nurses in mental healthcare institutions impact negatively on the provision of quality healthcare (Jack-Ide et al. 2018:2). Similarly, in South Africa, Sobekwa and Arumachallam (2015:5) found that an inadequate number of professional nurses have to deal with a huge number of MHCUs resulting from high admission rates and, therefore, nurses experience an unbearable workload. In the same study, it was further revealed that although both male and female nurses were exposed to threats of violence and assaults from MHCUs under their care, female nurses were sometimes helpless in the ward, and needed protection by the male nurses. A study conducted in the KwaZulu-Natal Province of South Africa revealed that psychiatric nurses working in acute psychiatric wards work under stressful situations, which results in increased levels of burnout and frustration. It was further revealed in the same study that nurses experience complex challenges in the psychiatric wards because they are always faced with aggressive and unpredictable patients (Joubert & Bhagwan 2018:54).

In South Africa, the deinstitutionalisation of mental healthcare has progressed at a rapid rate, as indicated in the 2013–2020 strategic plan; however, community-based services for MHCUs are still not well developed (Department of Health 2013:16). Therefore, the majority of MHCUs who are supposed to be receiving continuous care from the community-based settings are admitted to the psychiatric units of general hospitals and specialised psychiatric hospitals. Limpopo Province has only three specialised psychiatric hospitals, which provide care, treatment and rehabilitation for MHCUs. These specialised psychiatric hospitals also admit state patients and mentally ill prisoners according to section 41 and 49 of the *Mental Health Care Act* (Act no 17 of 2002). Although the 2013–2020 strategic plan declared to improve mental health for all by the year 2020, the environment in which nurses work is still swamped with complex challenges. Several studies were conducted on the challenges experienced by nurses in mental health institutions, but very few studies in South Africa have sought to understand the conditions under which professional nurses in specialised mental healthcare institutions are working. There is scant information on the circumstances under which professional nurses are working in specialised mental healthcare institutions in the Limpopo Province; therefore, it is important to unearth these circumstances, so that policymakers and the National Department of Health can be aware and act accordingly.

## Problem statement

Although the rights of the MHCUs are clearly stipulated in the *Mental Health Care Act* (Act no. 17 of 2002), measures to protect nurses working in the mental healthcare institutions are not clearly explained in the Act. Healthcare workers including nurses have an obligation to provide quality care to their patients without discrimination. However, nurses in the mental healthcare institutions struggle to control MHCUs who demonstrate violent behaviour. The authors of this article learnt with grief of the killing of a nurse by an MHCU who was admitted to one of the specialised psychiatric hospitals in the Limpopo Province. The nurse was killed on 14 April 2016 by an MHCU who was admitted for forensic observation in the hospital’s maximum security ward (Department of Health 2016).

In spite of the shortage of staff, little is known about the challenges that the nurses face in their day-to-day activities. Therefore, the aim of the study was to explore and describe challenges experienced by nurses working at a mental healthcare institution in the Limpopo Province.

## Objective of the study

The objective of the study was to explore and describe the challenges faced by the professional nurses working in a mental healthcare institution in Limpopo Province of South Africa.

## Definitions of key concepts

### Challenges

Challenges refer to difficulties that are encountered by people when they are in the process of making something to happen (Funk et al. 2018). In this study, challenges shall refer to difficulties experienced by professional nurses in a mental health institution whilst executing their duties.

### Professional nurse

According to the *Nurses Act* (Act no 33 of 2005), a professional nurse refers to a person registered in a category under section 31(1) in order to practise nursing or midwifery (South African Nursing Council 2005). In this study, a professional nurse shall refer to a qualified nurse registered with the South African Nursing Council to practice as a general nurse, midwife, psychiatric nurse or community nurse, and working at a mental health institution.

### Mental health institution

According to the *Mental Health Care Act* (Act no 17 of 2002), a mental health institution refers to a health establishment that provides care, treatment and rehabilitation services only for users with mental illness (South Nursing Council 2002).

### Mental healthcare user

According to the *Mental Health Care Act* (Act no 17 of 2002), an MHCU refers to a person receiving care, treatment and rehabilitation services, or using a health service, at a health establishment aimed at enhancing the mental health status of a user, state patient and mentally ill prisoner, and where the person is below the age of 18 years or is incapable of taking decisions, and in certain circumstances may include the person’s next of kin or a person authorised by any other law or court order to act on that person’s behalf (South Nursing Council 2002).

## Research method and design

A qualitative, exploratory and descriptive design was used to explore the challenges experienced by professional nurses at a mental health institution. This design made provision for obtaining in-depth information from professional nurses regarding the challenges that they experience when providing care to MHCUs.

## Study setting

The study was conducted at a selected hospital for MHCUs in the Limpopo Province of South Africa. The Limpopo Province is located at the northern part of South Africa, and it comprises five districts. There are only three specialised psychiatric hospitals that provide care to MHCUs in the province. This selected mental health institution provides services to three categories of MHCUs, namely, those with intellectual disability, those with chronic mental illness and forensic patients. The selected mental institution has a total number of 219 MHCUs and a total number of 65 professional nurses. The staff of the institution also include psychologists, occupational therapists and social workers. Most of the employees work in shifts because the mental health institution operates 24 h a day.

## Population and sampling

The population included both male and female professional nurses working at the selected mental health institution in the Limpopo Province. Purposive sampling was used to select professional nurses who were working in a mental health institution with MHCUs. The selection criteria required participants working at a selected mental healthcare institution for a period of not less than 3 years. The rationale for selecting nurses with 3 years of experience was because the study was qualitative, and we wanted people who were familiar with the institution and were able to describe their experiences. Data saturation occurred after interviewing 18 professional nurses (13 females and five males). The age of the participants ranged from 26 to 50 years.

## Data collection method

Data were collected using semi-structured individual interviews (Brink, Van der Walt & Van Rensburg 2018:144). The study was conducted from July 2016 to December 2016. An interview guide with predetermined questions was used to collect data from the participants by one of the authors who is a social worker by profession. He did not have a prior relationship with the participants because he did not work in the same institution as the participants. A central question to stimulate the discussion with the participants was asked: ‘can you please explain the challenges that you experience when providing care to mental healthcare users?’ Probing and follow-up questions were asked according to the responses from the participants. The interviews with the professional nurses were audio-recorded with the consent of the participants in order to capture the detailed discussion during the interviews. Field notes were used to document non-verbal cues from participants. Participants were interviewed in a private office at the mental health institution where they work during meal breaks. Data were collected using the English language, and the interviews lasted between 30 and 40 min. Probing and paraphrasing were used to get in-depth information and clarity from the participants. Data saturation occurred after 18 participants were interviewed. The interviews were transcribed verbatim to prepare for data analysis.

## Data analysis

The data were analysed using Tech’s method of open coding (De Vos 2011:403). All transcripts were read carefully to obtain a sense of the whole. Ideas were written down as they came to mind. The researcher selected one transcript from one interview and asked what this was about and reflected about the underlying meaning in the information. The researcher’s reflections were then written in the margin. A list was made of all themes, and similar themes were clustered together. The researcher applied the list of themes to the data. The themes, abbreviated as codes, were then written next to the appropriate segments of the transcript. Themes and sub-themes were developed and were verified.

## Trustworthiness

Trustworthiness was ensured through credibility, dependability, confirmability, comfortability and transferability. The activities to ensure credibility included member checking and prolonged engagement by going back to the participant for data verification, peer review by engaging supervisors and peers in research seminar presentations, and use of field notes to record non-verbal cues observed during data collection. Dense description of the research process and findings was used to enhance transferability. Dependability was utilised to ensure consistency, which was enhanced by coding and re-coding of data by one of the supervisors with experience in qualitative research. The strategy of conformability was used to ensure neutrality. Transcripts, audio-recordings and field notes were made available to supervisors to confirm the findings.

## Findings

Four themes emerged from data analysis, as shown in Table 1. These were: inadequate safety measures, inadequate resources, work-related challenges and shortage of staff. Themes were further divided into sub-themes such as violent behaviour of MHCUs, ineffective security measures, shortage of running water, poor condition of infrastructure, shortage of drugs, insufficient budget, stress, burnout, shortage of trained mental healthcare professional nurses and shortage of psychiatrists.

**TABLE 1:** Themes and sub-themes that emerged from the study.

Themes	Sub-themes
Inadequate safety measures	Violent behaviour of mental healthcare users
	Ineffective security measures
Inadequate resources	Shortage of running water
	Poor condition of infrastructure
	Shortage of drugs
	Insufficient budget
Impact of high workload	Stress
	Burnout
Shortage of staff	Shortage of trained mental healthcare professional nurses
	Shortage of psychiatrists

### Theme 1: Inadequate safety measures

Participants expressed a challenge of inadequate safety measures in the institution because they were working with MHCUs whose behaviour was unpredictable. The participants explained how unsafe they were when working in an institution that cares for MHCUs. Two sub-themes emerged from this theme, namely: violent behaviour of MHCUs and ineffective security measures.

#### Violent behaviour of mental healthcare users

The findings revealed the aggressive behaviour of MHCUs towards the nurses. One of the professional nurses explained that she was once assaulted by MHCUs under her care:

‘I was once hurt with a stone on the leg by a mental healthcare user. It just happens out of the blue and felt a pain. Some of the mental health users here have been referred by the court of law after committing a serious crime and they have access to move around the wards. Seriously, I don’t feel safe in caring for mental healthcare users.’ (P3, 50 years old, female)

Another participant stated:

‘We usually conduct prayer sessions in the morning together with mental healthcare users. To be honest with you I don’t close my eyes throughout the session to ensure that I can see whatever is happening there. The reason I don’t close my eyes is that you never know what will happen if you close your eyes.’ (P10, 48 years old, female)

#### Ineffective security measures

Participants expressed their fears when working in the mental healthcare institution. The findings revealed that participants were always in fear of being attacked by MHCUs at any time because there are no security personnel inside the wards to protect them from being hurt by their patients:

‘I don’t feel safe working in this environment. You know caring for mental healthcare users is very tough. You never know (shrugging shoulders) when and how will a mental healthcare user attack you. You just must stay alert and be careful. One moment he or she is fine and in the next moment he is very angry with you and you don’t know what to do.’ (P15, 28 years old, male)

‘There are few security officers which are placed in the main gate and corridors in a way that becomes difficult for a professional nurse working in the ward to be assisted as early as possible if the need arises.’ (P1, 36 years old, female)

### Theme 2: Inadequate resources

Professional nurses explained that there was a serious shortage of resources at the mental healthcare institution. The participants indicated that their concerns involved a shortage of running water, poor conditions of infrastructure, shortage of drugs and insufficient budget.

#### A shortage of running water

A shortage of running water at the hospital was regarded as very serious by participants. The findings further revealed that the institution was crippled by frequent water shortages. This resulted in disruption of critical services at the institution such as laundry and cooking. Two participants commented:

‘I have been working here for the past 10 years, the issue of water is worsening by each year.’ (P3, 50 years old, female)

‘… [*I*]n this hospital there is frequent water cuts which always delays cooking and laundry for mental health users.’ (P18, 32 years old, male)

#### Poor conditions of infrastructure

Participants were in despair about the poor conditions of the infrastructure, which they described as devastating. Participants stated that the environment in which they found themselves working was not conducive to their own health and that of their patients. It was revealed that the wards are in a state that it is not safe to accommodate patients because the buildings are very old and dilapidated:

‘We operate in an environment which I can’t call a psychiatric hospital.’ (P15, 28 years old, male)

‘The department should do something because some of the wards are no longer fit to house mental health users.’ (P8, 43 years old, female)

‘We are working in an infrastructure which is very old and not user friendly like other mental healthcare institutions. Wards are not enough to accommodate our patients, you know! How do we keep violent patients from others until they are stabilised?’ (P9, 30 years old, female)

#### Shortages of drugs

The findings revealed a lack of essential drugs to manage serious mental disorders. Professional nurses expressed concern that the shortage of drugs resulted in the relapse of patients:

‘At this hospital we deal with mental healthcare users with extreme psychotic conditions however most of the essential drugs which we requested are not always available even if we call the district management that there is no medication.’ (P7, 47 years old, female)

‘The majority of our patients relapse not because they don’t drink medication, it is because that sometimes their medication is not readily available, so we are forced to improvise. When they relapse, they tend to be violent.’ (P11, 33 years old, female)

#### Insufficient budget

Insufficient cash flow to run a psychiatric hospital was cited as a serious challenge. The majority of the participants explained that the hospital had serious cash flow challenges, hence the majority of the planned activities are not implemented. The following excerpts from participants are reported as follows:

‘The institution experiences financial problems especially the money to purchase essentials such as food and soap for the laundry.’ (P2, 31 years old, female)

‘Just imagine how we operate, the CEO of this institution has to borrow things like soap, cooking oil from other government institutions in the area.’ (P13, 29 years old, female)

### Theme 3: Impact of high workload

During the interviews, participants indicated that they were experiencing work-related stress and burnout because of high workload. Participants further expressed the lack of strategies to deal with the high workload that they were experiencing.

#### Stress

During the interviews, professional nurses expressed that they were experiencing work-related stress because of high workloads. A participant said:

‘It is very stressful to work in this environment and we are falling to cope. One of my colleagues has previous collapsed in the ward due to stress and she was also pregnant during the time. I think all this thing are happening perpetuated by the working condition that is not good at all.’ (P8, 43 years old, female)

#### Burnout

It was also revealed during the interviews that professional nurses were experiencing burnout as a result of high workload. Participants explained:

‘I can’t cope anymore. I feel emotionally exhausted I am going to ask for a leave or transfer to another department.’ (P3, 50 years old, female)

‘Working in this department is a prison sentence because the workload affects my life and is spilling into my social life.’ (P3, 50 years old, female)

### Theme 4: A shortage of staff

A shortage of staff was expressed as a challenge by professional nurses. The findings revealed a serious shortage of trained mental healthcare nurses and psychiatrists in the mental health institution.

#### A shortage of trained mental healthcare nurses

The majority of the participants in the current study expressed that there was a serious shortage of mental health nurses. It was further revealed that there were professional nurses who were failing to cope with work-related stress as a result of the shortage of professional nurses. Participants said:

‘We are not coping with the workload at hand and ever since we requested the management to hire additional staff, no response has been made. Usually we struggle to balance our leave register to ensure that the available nurses can manage their daily responsibilities.’ (P6, 40 years old, male)

#### A shortage of psychiatrists

The findings of the current study revealed a shortage of psychiatrists. Professional nurses explained that there is only one psychiatrist in their hospital who also works at other general hospitals in the district:

‘At the moment, we have only one psychiatrist, imagine… (quiet)…this is the only specialised psychiatric hospital in this district which also caters for forensic patients. I mean patients who have committed crimes.’ (P5, male)

### Ethical consideration

Ethical approval to conduct the study was obtained from the University of Venda, Research Ethics Committee (Ethical Clearance Number: SHS/16/PH/05/0905).

## Discussion

Findings revealed that the safety of professional nurses at their workplace was at risk, and some participants explained previous incidents of violence, which happened during interactions with MHCUs. The findings revealed that majority of the participants were females who also expressed their fear of being attacked by male MHCUs who are in the majority amongst the MHCUs. The findings are similar to those in a study on workplace violence in acute psychiatric settings of Northern Taiwan in which nurses were exposed to physical and psychological violence inflicted by their MHCUs (Niu et al. 2019:5). In the same study, it was further revealed that female nurses who work night shifts were the ones who were exposed to violent attacks by their MHCUs than those working fixed day shifts. This is consistent with a study conducted in Spain by Llor-Esteban et al. (2017:35), who revealed that professional nurses were exposed to risks of violence from MHCUs in their working environment. A study conducted in South African public mental health facilities by Sobekwa and Arunachallam (2015:5) also revealed that nurses experience aggression from MHCUs. In addition, a study conducted in mental health facilities in South Africa by Maluleke and Van Wyk (2017:9678) found that female psychiatric nurses experienced an unsafe work environment and sexual harassment during weekend shifts. The lack of security impacts on the execution of duties by professional nurses. Participants expressed their fear of being attacked by MHCUs who move freely around the ward. Participants further explained that some of the MHCUs were referred to their institution by the courts for observation. This was emphasised by Marshall, Craig and Meyer (2017:31), who also found that professional nurses in New Zealand are prone to violence in their workplace environment. Duncan et al. (2016:57) confirmed in their findings that professional nurses in Canada experience emotional and physical abuse from MHCUs.

The lack of running water in the psychiatric hospital is problematic because clean running water is essential for hospital sanitation. A study conducted by Bartram et al. (2015:210) found that one in three hospitals in developing countries do not have running water. Bartram et al. (2015:210) emphasised that enough water, sanitation and hygiene are vital components for providing a health service. The consistent provision of water in hospitals helps to avert outbreak of deadly diseases, which can affect patients and healthcare workers. Interrupted supply of water at the mental health institution affects the quality of provision of healthcare services to MHCUs because it affects the preparation of meals and hygiene, hence chances of contracting other diseases. Delays in provision of meals may affect the intake of medication by MHCUs, resulting in delayed recovery and risk of relapsing.

The participants described the environment as worn out and not conducive for working. The national study conducted in South Africa also revealed that the insufficient infrastructure in public mental health care institutions resulted in MHCUs being kept with other patients in over 80% of district hospitals in Limpopo, KwaZulu-Natal, Mpumalanga and Northern Cape provinces. (Docrat, Besada, Cleary, Daviaud & Lund 2019:714). This was also confirmed by Manyisa and Van Aswegen (2017:34) who identified poor physical conditions of the buildings, wards and the general layout of psychiatric hospitals as challenges to the provision of mental healthcare. In a study conducted in Nigeria, Nwaopara, Abasiubong and Umoh (2016:64) found that unsuitable environment exposes nurses to physical and emotional harm that hinder the provision of psychiatric care. The poor condition of mental healthcare facilities in South Africa is attributed to decades of underfunding, which results in dilapidated buildings unfit for use (Van Rooyen et al. 2019:2). Even though there is insufficient evidence to suggest that the physical environment enhances mental treatment outcomes, the findings of a study conducted in Tanzania has revealed that a conducive environment assists patients to recover, as it is considered a pure therapeutic environment (Ambikile & Iseselo 2017:17). Therefore, governments in developing countries, including South Africa, should strive to prioritise mental health in order to improve treatment outcomes (Sunkel & Viljoen 2017:15). The findings revealed that poor conditions of the buildings may hinder the MHCUs’ recovery process because the environment is not attractive and therefore does not promote calmness. It was further revealed that there were no private rooms and therefore the privacy of MHCUs was compromised because of the disruptions that come with the sharing of rooms. A study conducted in the United Kingdom suggests that mental health institutions should be environments of healing that allow the building itself to be part of the therapeutic setting and process (Zhang, Tzortzopoulos & Kagioglou 2019:747).

It was further revealed that shortages of drugs in a mental health institution expose healthcare workers to extreme violence from MHCUs. Similar findings were reported in other studies in South Africa, whereby the mental healthcare system is in dire straits with shortages noted in psychiatric medications, especially in rural public health facilities (Sunkel & Viljoen 2017:16). A study conducted in Mozambique reported that psychiatric medications on the essential list were not always available, for example, typical antipsychotics and fluoxetine, in national and district mental health facilities (Wagenaar et al. 2015:279). Another study conducted in a Tanzanian psychiatric hospital reported that there was a lack of essential drugs for MHCUs. The unavailability of the essential psychotropic drugs was attributed to poor procurement practices and budget cuts because of competing demands, which delayed steady supply of the medication (Iseselo & Ambikile 2017:16). Similar findings were reported in Ghanaian mental health institutions, where rampant corruption and bureaucracies delayed the constant supply of medication (Oppong et al. 2016:28). The shortage of mental health drugs affects the provision of quality of care, hence there is a huge treatment gap in Africa (Iseselo & Ambikile 2017:16). According to Monteiro (2015:95), African countries spend less on mental healthcare as compared to other competing needs. For example, Nigeria spends less on mental healthcare (only 4% of the gross domestic product). Therefore, the majority of African governments dedicate less than 1% for mental healthcare. According to Lund et al. (2012:404), in South Africa, ‘the resources required to deliver mental health services including human resources, service facilities and budgets have been consistently inadequate’. Furthermore, Docrat et al. (2019:706) suggested that the inadequate budget for mental healthcare in South Africa is a by-product of low prioritisation of mental health issues in the country.

Professional nurses were experiencing stress and burnout resulting from work-related challenges. They further explained that they have lost passion for their profession because of work-related stress. A study conducted in Nigeria by Hanson, Onasoga and Babalola (2017:28) confirmed that the workplace environment plays an integral part in the causation of stress because people spend most of their time in the work setting. It was indicated that professional nurses caring for MHCUs are prone to substance abuse because of the scope of their duty. It is also stated that some of the professional nurses suffer from headaches, sleep disturbances, chest pains and other related symptoms of stress (Hanson et al. 2017:34). A study conducted in the United States by Edward, Hercelinsky and Giandinoto (2017:215) emphasised that there is a robust relationship between high levels of emotional exhaustion amongst professional nurses working in mental health institutions and high workload being experienced in the workplace. In addition, similar findings were reported by Laker et al. (2019:190), who reported that nurses in a United Kingdom hospital were prone to burnout and emotional exhaustion. In the current study, participants expressed emotional exhaustion resulting from an increased workload, which may affect the provision of healthcare.

The shortage of professional nurses has resulted in high workloads and work-related stress for the available professional nurses. Participants explained that they were unable to balance their leave register because of the shortage of trained mental healthcare professional nurses. Therefore, professional nurses at the mental health institution are overworked. A study conducted by Hlongwa and Sibiya (2019:2) revealed that in KwaZulu-Natal, there were shortages of nurses with advanced psychiatric nursing. Alburquerque-Sendin et al. (2018:293) confirmed that resources are very limited in the South African mental healthcare institutions, although they are very important in the provision of quality mental health service. This can be attributed to a brain-drain, and the flight of skilled personnel to rich countries, which is occurring in African countries (De Kock & Pillay 2018:124). Mental healthcare institutions within the continent of Africa are not fully equipped to cater to the needs of mental health users, and the environment is not conducive for professional nurses (Oleribe et al. 2019:395). A study by De Kock and Pillay (2017:4) indicated that there is a serious shortage of mental health professionals, including nurses in South Africa, and, hence, it impedes the provision of quality care in all facets of healthcare including mental healthcare.

The participants explained that shortage of critical staff such as psychiatrists derails the provision of mental healthcare. This finding is consistent with a study conducted by De Kock and Pillay (2018:124), which concludes that there was a serious shortage of psychiatrists in South African rural mental health facilities. Furthermore, another study, which was conducted in South African public hospitals, revealed that there are very few psychiatrists (0.03 per 100 000) in rural healthcare facilities (De Kock & Pillay 2017:1). According to the World Health Organization, about 0.5 psychiatrists per 100 000 population are required to manage medium-stay residential centres, acute in-patient care and an out-patient and primary care centre (Docrat et al. 2017:709). De Kock and Pillay (2017:4) reported that South Africa is challenged by the failure to provide human resources for mental healthcare, whereby the majority of the psychiatrists operate in private institutions. The shortage of psychiatrists delays the recovery of MHCUs.

### Limitations

The study was conducted in only one mental health care institution using the qualitative method of data collection, and the sample was confined to only one mental health care institution. Because the study was confined to only one group of healthcare workers in a specialised psychiatric hospital, it could not explore the challenges that confront other categories of healthcare workers. Therefore, a quantitative study with a large sample should be considered in future to give a clear picture of the challenges that affect professionals working in mental health care institutions.

### Recommendations

The study recommends that the government should review the *Mental Health Care Act* no 17 of 2002 because it focusses on the rights of MHCUs, which include the right to dignity and free movement, and it does not mention anything about the rights and safety of professional nurses and mental health care professionals. Furthermore, it is recommended that there should be active intersectoral collaboration between the health sector, correctional services and other departments involved in the planning and implementation of mental health services. To protect the mental health care professional, the management of the facility should notify the Department of Health about the need to hire security officers who will provide their services directly in the ward. In addition, the risk management office should conduct workshops and training on management of aggressive behaviour of the MHCUs. The buildings and emergency exits should be functional to ensure a safe and therapeutic environment, especially in cases of emergency. More so, professional nurses should be exposed to employee wellness programmes to enhance their coping strategies regarding work-related stress and burnout. The Department of Health should allocate resources that are necessary for the proper functioning of the mental health care institution.

## Conclusion

The results of this study reveal complex challenges experienced by professional nurses when providing health care services to MHCUs in the mental health institutions. The findings revealed safety risks as a serious concern amongst professional nurses in specialised mental health institutions. The study revealed that there is a serious shortage of human and material resources, which also affect professional nurses emotionally because they cannot provide health care services effectively whilst under stress. The working conditions in the mental health care institutions should be conducive to promote the provision of quality health care to MHCUs by professional nurses and other health care team members. Most of the challenges expressed in this study are affected by the budget and therefore it needs government interventions.
